# Temporal trends and educational inequalities in obesity, overweight and underweight in pre-pregnant women and their male partners: a decade (2010–2019) with no progress in Sweden

**DOI:** 10.1093/eurpub/ckae052

**Published:** 2024-03-20

**Authors:** Thomas Vogt, Marie Lindkvist, Anneli Ivarsson, Sven-Arne Silfverdal, Masoud Vaezghasemi

**Affiliations:** Department of Epidemiology and Global Health, Umeå University, Umeå, Sweden; Department of Epidemiology and Global Health, Umeå University, Umeå, Sweden; Department of Epidemiology and Global Health, Umeå University, Umeå, Sweden; Department of Clinical Science, Pediatrics, Umeå University, Umeå, Sweden; Department of Epidemiology and Global Health, Umeå University, Umeå, Sweden

## Abstract

**Background:**

Trends in overweight and obesity among expectant parents can provide useful information about the family environment in which children will grow up and about possible social inequalities that may be passed on to them. Therefore, we aimed to assess whether the prevalence of underweight, overweight and obesity changed over time in pre-pregnant women and their male partners in northern Sweden, and if there were any educational inequalities.

**Methods:**

This study is based on cross-sectional data from a repeated survey of the population in Västerbotten, Sweden. The study population included 18,568 pregnant women and 18,110 male partners during the period 2010–2019. Multinomial logistic regression models were fitted separately for pregnant women and male partners to assess whether the prevalence of age-adjusted underweight, normal weight, overweight and obesity had evolved between 2010 and 2019, and whether trends differed by educational level.

**Results:**

Among women, obesity prevalence increased from 9.4% in 2010 to 11.7% in 2019. Among men, it went from 8.9 to 12.8%. Educational inequalities were sustained across the study period. In 2019, the prevalence of obesity was 7.8 percentage points (pp) (CI = 4.4–11.3) higher among women with low compared to high education. The corresponding figure for men was 6.4 pp (CI = 3.3–9.6).

**Conclusions:**

It is not obvious that the prevalence of obesity among parents-to-be will decrease under current dispositions. Public health policies and practice should therefore be strengthened.

## Introduction

High body mass index (BMI) is associated with disability and death from cardiovascular diseases, type 2 diabetes, chronic kidney disease and cancers.^1^ Alarmingly, the prevalence of overweight and obesity has been increasing for decades in all regions of the world, for both adults^2^ and children.^1^ It is estimated that close to 40% of the world’s adult population is overweight or obese.^2^ However, there is some evidence that the prevalence of overweight and obesity in adults might have stopped increasing in some countries such as the United States and the United Kingdom.^2^ This is in line with a proposed framework of the obesity transition suggesting that the on-going obesity pandemic will finally move over to a period with decrease in obesity prevalence, starting with the high-income countries.^3^

In Sweden we are not aware of any reports of such a shift to a decrease in obesity prevalence over time. In contrast, studies in Sweden have reported an increased prevalence of overweight and obesity among adults in the last decades and up to 2017, with no apparent sign of levelling off.^4^ For pre-pregnancy overweight and obesity, an overall increase was observed from 1992 to 2010,^5^ and from 1982 to 2013,^6^ Specifically for the region of Västerbotten in northern Sweden, the age-adjusted prevalence of pre-pregnancy overweight and obesity combined increased from 20.4 to 35.7% during that time period, while the prevalence of obesity increased from 5.2 to 10.8%.^5^ Signs of a slowing-down of the increase in mean BMI and obesity prevalence among middle-aged individuals has however been observed in the region over the period 1990–2007, but trends differed based on gender, living area and socioeconomic status.^7^

Despite Sweden’s reputation as a Welfare State and as a relatively equitable society, the prevalence of overweight and obesity has been higher in socioeconomically disadvantaged groups at least since the late 1960s.^8^ Although it has been thought that socioeconomic inequalities in obesity have remained stable over time, recent evidence suggests that absolute inequalities have been widening among pre-pregnant women to the disadvantage of those with lower education,^6^^,^^9^ and among 18 to 19 years old men to the disadvantage of those with parents of lower education.^10^ Similarly, the slowing-down observed in Västerbotten was mostly observed in university-educated individuals living in urban areas but not in other groups, and the gap between groups with higher compared to lower education seemed to have increased over time.^7^

Additional considerations when it comes to the adults’ BMI are that maternal obesity prior to conception is strongly associated with overweight and obesity in children,^11^ and, overall, the BMI of both parents appears to matter for children overweight and obesity.^12–14^ Both parents are, therefore, important actors for the prevention of childhood overweight, and for the fulfilment of the World Health Organization’s goal of ‘no increase in childhood overweight’ by 2025.^15^ Thus, monitoring time trends of overweight and obesity in parents-to-be can provide useful information concerning the environment in which children will be growing up, and on potential social inequalities in the parents’ BMI that may be passed on to children. This may be particularly important in anticipating future needs for overweight/obesity prevention since in Europe—Sweden included—a socioeconomic gradient similar to the one found in adults has been observed for children of school age.^16^^,^^17^

### Aims

We therefore aimed to assess whether the prevalence of underweight, normal weight, overweight and obesity changed over time in pre-pregnant women and their male partners in northern Sweden, and if there were any educational inequalities.

## Methods

### Study setting and study population

This repeated cross-sectional study used data routinely collected in Antenatal Care as part of the Salut Child Health Promotion Programme, an ongoing universal health promotion programme in the region of Västerbotten, northern Sweden. Prior to visiting Antenatal Care around the 11th week of gestation, pregnant women and their partners were invited to fill out a questionnaire that included items related to educational attainment, family situation, health, living conditions and lifestyle habits. Two items were enquiring about the respondents’ weight and height, thereby allowing the calculation of their BMI (kg/m^2^).

We used questionnaires collected between 2010 and 2019, covering a decade. In this study, our two target populations were women who lived in Västerbotten and became pregnant during that decade, and their male partners. Female partners were not considered, as they were too few.

The study sample (*n* = 36 678) was therefore composed of those pregnant women and male partners who filled out a questionnaire prior to the routine visit to Antenatal Care. From 2010 to 2019, there were 29 047 live births in Västerbotten (Supplementary table S1)^18^ and 42 249 questionnaires were filled out by pregnant women and their male partners. Among those 42 249 questionnaires, 2566 did not consent for the information to be used in research and were therefore not included in this study. Another 3 were excluded because their reported BMI was superior to 60. In addition, 3002 observations had missing data on BMI, age, or both, and were also excluded. Therefore, the final sample size included 18 568 pregnant women and 18 110 male partners. This suggests that the study sample comprised 64% of all women who became pregnant in Västerbotten during the study period. When considering educational attainment in addition to age and BMI, 573 individuals with missing information on educational attainment were also excluded, leaving *n* = 18 215 pregnant women and *n* = 17 890 male partners for analysis.

### Measures

*BMI* was calculated from the weight and height reported in the questionnaire around the 11th week of gestation, as kg/m^2^. It could not be validated against more objective measurements. For male partners, *BMI* was the current BMI when filling out the questionnaire. For pregnant women, however, it was the BMI as it was just before the pregnancy.

*Age-adjusted BMI* was obtained for pregnant women by regressing *BMI* on age and on a quadratic term for age, and by using the predicted BMI-values from the model. For male partners, the procedure was similar but no quadratic term for age was used in the linear regression model. This method has been used in the previous BMI research,^19^^,^^20^ and we used two different regression models to achieve age-adjustment due to differing patterns observed in the data in men and women when it came to the relationship of BMI with age.

*BMI* and *age-adjusted BMI* were further categorized into (i) underweight (BMI < 18.5), (ii) normal weight (18.5≤ BMI <25), (iii) overweight, excluding obesity (25 ≤ BMI <30) and (iv) obesity (BMI ≥30) to create two variables: *BMI categories* and *age-adjusted BMI categories*. However, these cut-offs apply only to adults aged 18 years-old or more, and some individuals in the sample were under 18 (*n* = 23). To categorize them, we applied the Extended International (IOTF) Body Mass Index Cut-Offs for Thinness, Overweight and Obesity in Children.^21^

To calculate prevalence ratios (PRs) and prevalence differences (PDs), *age-adjusted BMI* was also dichotomized into (i) obese, and (ii) not obese; as well as (i) overweight (excluding obesity) and (ii) not overweight -for both men and women. For women only, it was also dichotomized into (i) underweight and (ii) not underweight.

Educational attainment was recorded in the questionnaire as the highest level of education that the respondent had completed and included: (i) less than 9 years of schooling, (ii) compulsory school or the equivalent of 9 years of schooling and (iii) secondary school or the equivalent of 12 years of schooling, (iv) post-secondary education, less than 3 years, or (v) post-secondary education, 3 years or more. Because only a small number of participants fell into categories (i) and (ii) - and (iv) to a lesser extent—education was further dichotomized into: (i) low education (completion of secondary school or less), and (ii) high education (completion of at least some post-secondary education).

### Statistical analysis

Descriptive statistics for BMI and age is displayed in Supplementary table S1, with the corresponding number of live births in Västerbotten per year. The prevalence of each BMI category for each year is displayed in Supplementary table S4 for unadjusted BMI and in Supplementary table S1 for age-adjusted BMI. Numbers and percentages are presented separately for pregnant women and male partners. Supplementary table S2 presents the age-adjusted prevalence of BMI categories by educational attainment and year for pregnant women and male partners.

**Figure 1:**
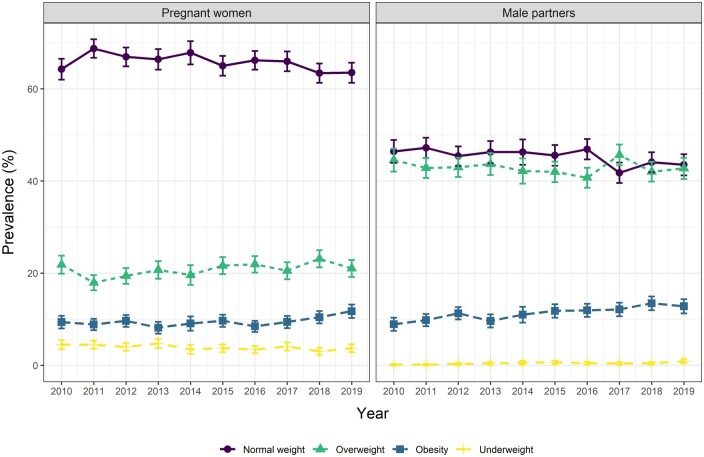
Age-adjusted prevalence of normal weight, overweight, obesity, and underweight in pregnant women (*n* = 18 568) and male partners (*n* = 18 110) from 2010 to 2019, with 95% CI estimated from multinomial logistic regression

**Figure 2:**
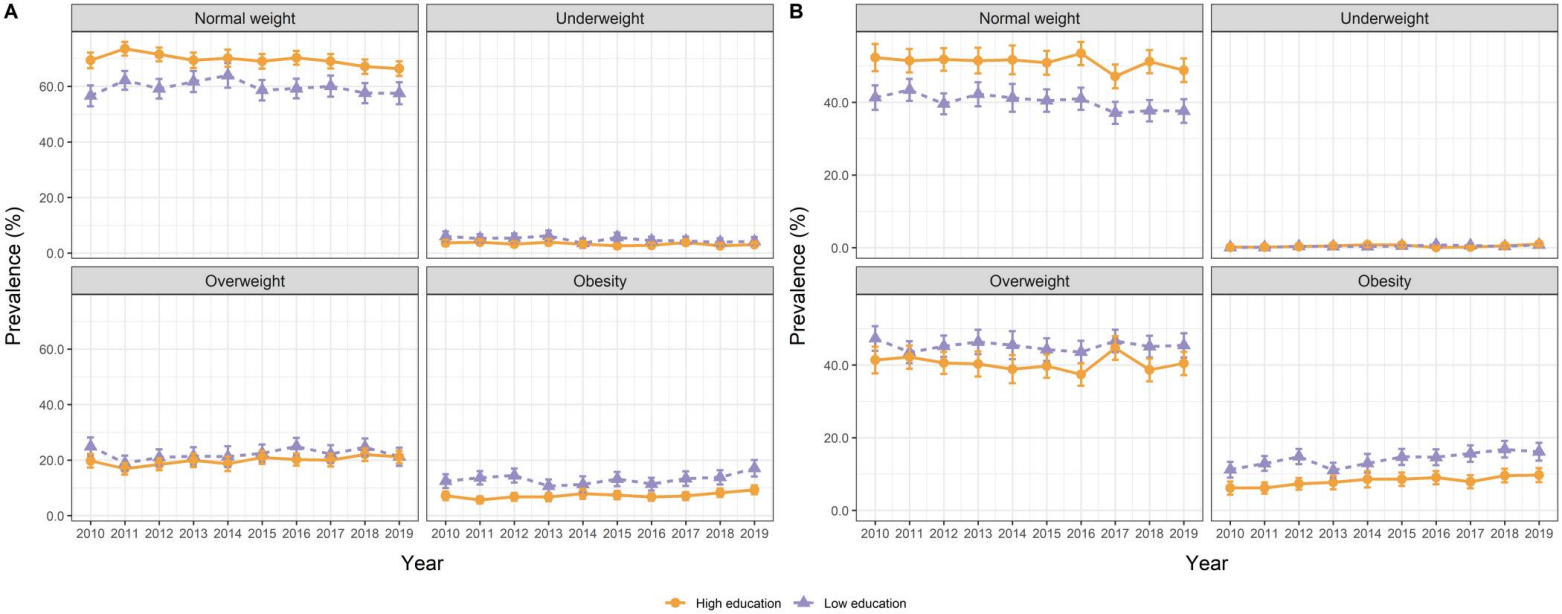
(A) Age-adjusted prevalence of normal weight, underweight, overweight, and obesity by level of educational attainment in pregnant women (*n* = 18 215) from 2010 to 2019. (B) Age-adjusted prevalence of normal weight, underweight, overweight, and obesity by level of educational attainment in male partners (*n* = 17 890) from 2010 to 2019. The 95% CI were estimated from multinomial logistic regression.

Multinomial logistic regressions with ‘normal weight’ as the reference category were fitted separately for pregnant women and male partners to assess whether the prevalence of age-adjusted BMI categories had changed between 2010 and 2019 for those groups. The year was treated as a categorical variable with 2010 as the reference (Supplementary tables S5 and S6). A further set of models was then fitted with year, educational attainment, and an interaction term of year*educational attainment as the independent variables (Supplementary tables S7 and S8). Here, 95% confidence intervals (CI) for the annual prevalence of each BMI category were then obtained from the multinomial regression models. In Stata, the margins command can be used for this purpose. Results were plotted in figure 1 separately for women and men, and in figure 2A (women) and figure 2B (men) were stratified by educational attainment.

Finally, PR and prevalence differences (PD) comparing the group with low education to the group with high education were estimated for each year for obesity and overweight (women and men), and underweight (women). This was done by regressing the binary versions of age-adjusted BMI on educational attainment, using generalized linear models with a binomial family distribution and a log link for the PRs, and an identity link for the PDs (Supplementary table S3).^22^ PDs are presented in pp.

The analyses were performed using R version 4.2.0 (the R Foundation for Statistical Computing) and Stata/BE version 17.0 (StataCorp, College Station, Texas 77845, USA).

### Ethics

The Swedish Ethical Review Authority approved the study (2010-63-31M). Only those who had given written informed consent were included in this study.

## Results

Over the study period (2010–2019), the annual mean BMI of pre-pregnant women was always lower than that of male partners and it stayed relatively stable for both groups, although a slight increase could be seen towards the end of the study period (Supplementary table S1). An overall 1.3% increase from 2010 to 2019 was observed for women (from 23.9 kg/m^2^ in 2010 to 2024.2 kg/m^2^ in 2019), and a 1.6% increase for men (from 25.7 to 26.1 kg/m^2^). The mean age also remained stable over the study period, except for the last year when it increased slightly for both men and women. In 2019, the mean age was 30.5 years for women and 32.7 for men.

Whether considering unadjusted or age-adjusted BMI (Supplementary tables S1 and S4), the prevalence of underweight, normal weight, overweight and obesity were about similar. We will therefore focus on age-adjusted BMI categories from here on. Most women across the study period reported a normal weight (63.5% in 2019), followed by overweight (21.0%), obese (11.7%) and underweight (3.7%). For men, however, the prevalence of normal weight and overweight was similar (43.5% and 42.7%, respectively, in 2019). Men with obesity formed the third largest group (12.8% in 2019) and men with underweight the smallest group (0.9% in 2019). Underweight individuals were few over the study period, especially among men. This precluded us from drawing conclusions on the evolution of underweight over time for male partners and its relation to educational attainment.

For women, only a small increase in obesity prevalence for the last two years of the study period was noticeable (figure 1). In the multinomial regression model including only *Year* as independent variable, the odds of being in the obese group relative to the normal weight group were only significantly higher in 2019 compared to the reference year of 2010 (OR = 1.27, CI=1.02-1.58) (Supplementary table S5).

The prevalence of obesity in men, however, showed an increasing trend throughout the study period (from 8.9% in 2010–12.8% in 2019), with corresponding small and inconsistent decreasing trends in the prevalence of normal weight and overweight (figure 1, Supplementary table S1). In the multinomial regression model including only *Year* as independent variable, the odds of being in the obese relative to the normal weight group were significantly higher in the second part of the study period, compared to the reference year of 2010 (for 2019: OR = 1.53, CI=1.21–1.94) (Supplementary table S6).

The prevalence of overweight and obesity was higher among women and men with lower educational attainments across the study period, except for 2019 for overweight among women (figure 2A and B; Supplementary table S2). In addition, the prevalence of underweight was systematically higher among women with lower compared to higher educational attainment. Accordingly, the PRs and PDs for overweight and obesity (for women and men) and underweight (for women) indicated a disadvantage for the lower educated groups throughout the study period (Supplementary table S3). In 2019, women in the lower educated group were 1.83 (CI = 1.43–2.35) times more likely to be obese than women in the higher educated group, corresponding to a 7.8 pp (CI = 4.4–11.3) PD in obesity between those two groups. The same year, men in the group with lower educational attainment had a 66% (PR = 1.66, CI = 1.30–2.14) higher prevalence of obesity than men in the group with higher educational attainment. This corresponded to a PD of 6.4 pp (CI = 3.3–9.6) between the two groups.

However, no specific trend for lower vs. higher educational attainment in the prevalence of underweight, overweight or obesity could be observed (figure 2A and B). In addition, in the multinomial regression model including year, educational attainment and the interaction term year*educational attainment as independent variables, no significant interaction between year and educational attainment was found (Supplementary tables S7 and S8).

## Discussion

In this repeated cross-sectional study of expectant parents in Västerbotten, the prevalence of obesity in 2019 was 11.7% among pregnant women, and 12.8% in male partners. A sustained increase in the prevalence of obesity was observed for men, but not for women, although the last two years of the study period could indicate a worrying trend that should be monitored in the future. The temporal trends did not significantly diverge between groups with different educational attainments. And indeed, a graphical examination of the temporal trends does not suggest that the difference in prevalence between groups with low vs. high education attainments is either widening or narrowing (figure 2A and B). However, the groups with lower educational attainment had a considerably higher prevalence of overweight and obesity across the study period, both for women and men, and a higher prevalence of underweight for women.

Despite the weight and height of participants in this study being self-reported, we found similar results to previous studies using measured weight. Chaparro et al. found the age-standardized prevalence of obesity and overweight (here excluding obesity for comparison purposes) among pre-pregnant women in Västerbotten to be 10.8% and 24.9%, respectively.^5^ The corresponding prevalences were 9.4% and 21.9% in our study (Supplementary table S1). Similarly, Lundberg, et al. reported a 10.5% prevalence of age-adjusted obesity in early pregnancy in 2013 in Sweden,^6^ when we found a prevalence of 8.2%, but in pre-pregnant women. Conversely, Hemmingsson et al. reported an unstandardized prevalence of obesity of 14.4% among women and of 18.1% among men in 2016–2017.^4^ However, the mean age in their study was 10 years higher than in the current study.

Chaparro et al. and Hemmingsson, et al. found a persisting increase in the prevalence of overweight and obesity from 1992 to 2010 and from 1995 to 2017, respectively.^4^^,^^5^ In Västerbotten, this corresponded to a more than two-fold increase in obesity prevalence from 1992 to 2010 in pre-pregnant women.^5^ In contrast, our study found a more modest increase in the prevalence of obesity across the study period for men, and only at the end of the study period for women. This could indicate a sustained slowing down of the obesity epidemic in the region, which has also been suggested by previous research.^7^

Like in the previous research, we observed educational inequalities in overweight and obesity^4^^,^^6–9^ disfavouring the groups with lower educational attainments. In addition to previous findings however, we also observed inequalities in underweight in women, with the prevalence of underweight being higher in the group with lower education attainments. Unlike the previous studies,^6^^,^^7^^,^^9^^,^^10^ we did not find signs of an increase in the PDs between groups with different educational attainments.

In short, it does not seem that Sweden is experiencing any levelling off in the prevalence of overweight and obesity in adults. An additional concern is that social inequalities, here reflected by educational attainment, might be persisting among adults in the country, with no sign of the gap becoming narrower.

### Implications for research and policy

This study should be updated when data for the following years becomes available, as we observed worrying signs that the prevalence of obesity might have been increasing over the study period. The impact of the COVID-19 pandemic on obesity trends in Västerbotten should also be examined.

In addition to considering the prevalence of predefined categories, further research on pregnant women and their partners in Västerbotten could consider temporal changes and inequalities across the entire distribution of BMI, as there is evidence that temporal trends and social inequalities may be more alarming at higher BMIs.^19^^,^^20^^,^^23^^,^^24^ The higher prevalence found of both overweight/obesity and underweight in the group of women with lower education attainment also suggests that social inequalities in BMI could be manifested at both ends of the distribution. Furthermore, other social factors could be considered. We will do so in future work, and we will obtain other data to complement the present work.

Our study also highlights the need for existing policies that can reduce the prevalence of overweight and obesity in the current adult population, prevent it for the next, and reduce inequalities, to be strengthened, or for new policies to be designed. Indeed, even in a country like Sweden, the prevalence of—and inequalities in—overweight/obesity are persistent and will likely not improve automatically.^3^^,^^8^

### Strengths and limitations

Our study period includes recent years and therefore expands existing research on adult’s BMI in Sweden.^4–10^ Another strength of this study is the inclusion of the partners of the mothers-to-be, although we could not include female partners due to the very small size of this group. Because all parents are thought to be relevant actors for childhood overweight and obesity, monitoring not only the BMI of pregnant women, but also of their partners, could be relevant for the monitoring of the obesity epidemic.^12^

Selection bias due to survey or item non-response might have affected the estimation of the prevalence of overweight/obesity and underweight, and of educational inequalities. For example, selection bias could have led to the underestimation of inequalities between groups with lower vs. higher educational attainment, if those with both lower educational attainment and high BMI were less likely to respond to the survey or to specific items.^25^ However, it is unlikely that selection bias alone would invalidate our main findings.

Reporting bias is another concern, as height and weight, educational attainment, and age were self-reported. Here, we will focus on the misreporting of BMI (height and weight), but bias could also be an issue for the reporting of age and education even though it is less likely. Although there can be a high variability in misreporting, underreporting of BMI is thought to be common in studies using self-reported measures of height and weight, as height tends to be overreported and weight tends to be underreported.^26^ In addition, the underreporting of BMI could be partly determined by any of the other variables of interest in this study, including true BMI.^27^ Individuals with obesity could therefore be more likely to overreport their height and underreport their weight compared to individuals with a normal weight, and conversely, men with underweight could overreport their weight.^28^ An additional challenge could be posed if reporting bias evolved over time. For example, if underreporting was present but decreased over the study period due to more accurate reporting, then it could explain the upward trend in male partners’ BMI and obesity prevalence.^27^ Furthermore, reporting bias might also affect the estimation of educational inequalities in obesity, overweight, or underweight, if there are different patterns of reporting bias in groups with different educational attainment.^29^

In addition, the small number of men with underweight in our sample prevented us from assessing the evolution over time of this specific category. Much larger samples are likely to be needed to describe trends in underweight among men in Sweden, as they are a very small group.

## Conclusion

The high prevalence of overweight and obesity among expectant parents in Västerbotten is a threat to public health and to the health of children to come. In this study, we saw an increase over time in the prevalence of obesity—mostly for men—that did not differ based on educational attainment. However, we observed educational inequalities that were sustained across the study period not only for obesity—and overweight to a lesser extent—but also for underweight for women. This highlights the need to consider the entire BMI distribution in future research and not only its mean or BMI categories. Finally, our results suggest that to reduce the prevalence of adult obesity in Västerbotten, public health policies and practice should be strengthened, as it is not evident that the obesity prevalence will decrease under current dispositions.

## Supplementary Material

ckae052_Supplementary_Data

## Data Availability

The datasets presented in this article are not readily available because Region Västerbotten originally collected the data for a child health survey (https://www.regionvasterbotten.se/salut). We accessed data for the present study after approval from both the Region Västerbotten and the Ethical Vetting Board. The data are not publicly available but access for replication analyses is possible. Requests to access the datasets should be directed to https://www.regionvasterbotten.se/salut. Key points We provide evidence of an increase in obesity prevalence among prospective parents during the decade 2010–2019. We also observed that the prevalence of overweight, obesity, as well as underweight was higher among the group with lower education compared to high education. We found that educational inequalities were sustained across the study period. Temporal changes and social inequalities across the entire distribution of BMI should be evaluated, as evidence suggests that they may be more pronounced at higher levels of BMI. It is not obvious that positive change will occur under current public health policies and healthcare practices, unless these are strengthened.
