# Supplementary material for: Temporal trends and educational inequalities in obesity, overweight and underweight in pre-pregnant women and their male partners: a decade (2010–2019) with no progress in Sweden
Source: Eur J Public Health. 2024 Mar 20;34(5):943–8. doi: 10.1093/eurpub/ckae052 (PMC11430927; doi:10.1093/eurpub/ckae052)
Supplement: ckae052_Supplementary_Data [file ckae052_supplementary_data.pdf]

**SUPPLEMENT TO: Temporal trends and educational inequalities in obesity, overweight, and underweight in pre-pregnant women and their male partners: A decade (2010-2019) with no progress in Sweden.**

**Contents**


**General characteristics of Västerbotten** .......... 2
**Table 1** .......... 3
**Table 2** .......... 7
**Table 3** .......... 11
**Table 4** .......... 15
**Table 5** .......... 17
**Table 6** .......... 19
**Table 7** .......... 20
**Table 8** .......... 22

**General characteristics of Västerbotten**

Västerbotten County is located in the northern part of Sweden around latitude 64o. The population increased from 259,286 in 2010 to 271,736 in 2019, with a density of 4.9 / km2. The immigration and emigration varied in this period between 2,200 and 3,500, and 800 and 1,100 persons respectively. The main part of the population lived in the cities of Umeå and Skellefteå with a higher educational level compared to the rural areas.

**Table 1: Characteristics of the sample (n=36,678) and live births in Västerbotten (n=29,047), from 2010 to 2019.**

| | Pregnant women (n= 18,568) | | | | | | | | | |
|---|---|---|---|---|---|---|---|---|---|---|
| **Years (n)** | **2010** (n=1716) | **2011** (n=2063) | **2012** (n=2020) | **2013** (n=1725) | **2014** (n=1321) | **2015** (n=1891) | **2016** (n=2067) | **2017** (n=1880) | **2018** (n=2011) | **2019** (n=1874) |
| **BMI (unadjusted)** | | | | | | | | | | |
| Mean (SD) | 23·9 (4·4) | 23·7 (4·4) | 23·8 (4·5) | 23·7 (4·3) | 23·8 (4·2) | 23·9 (4·5) | 23·9 (4·3) | 23·9 (4·4) | 24·3 (4·8) | 24·2 (4·5) |
| Median | 22·9 | 22·7 | 22·7 | 22·8 | 23·0 | 22·9 | 22·9 | 22·9 | 23·2 | 23·1 |
| (Min, Max) | (13·3, 49·1) | (14·7, 51·1) | (13·3, 54·5) | (16·2, 47·2) | (15·6, 47·0) | (14·4, 59·5) | (14·6, 47·4) | (15·9, 56·4) | (14·8, 59·5) | (15·8, 45·6) |
| **Age** | | | | | | | | | | |
| Mean (SD) | 29·8 (5·0) | 29·9 (5·0) | 29·7 (4·9) | 29·8 (4·9) | 29·8 (4·7) | 29·8 (4·8) | 30·0 (4·7) | 30·1 (4·6) | 30·0 (4·6) | 30·5 (4·4) |
| Min, Max | 17·0, 47·0 | 16·0, 46·0 | 16·0, 54·0 | 16·0, 47·0 | 18·0, 50·0 | 17·0, 47·0 | 16·0, 45·0 | 15·0, 45·0 | 17·0, 45·0 | 18·0, 47·0 |
| **BMI categories (age-adjusted)** | | | | | | | | | | |
| Underweight | 77 (4·5%) [a] | 92 (4·5%) | 81 (4·0%) | 82 (4·8%) | 46 (3·5%) | 70 (3·7%) | 71 (3·4%) | 77 (4·1%) | 61 (3·0%) | 70 (3·7%) |

| | | | | | | | | | | |
|---|---|---|---|---|---|---|---|---|---|---|
| (n=727) | | | | | | | | | | |
| Normal Weight (n=12,216) | 1103 (64·3%) | 1418 (68·7%) | 1352 (66·9%) | 1145 (66·4%) | 896 (67·8%) | 1229 (65·0%) | 1368 (66·2%) | 1240 (66·0%) | 1275 (63·4%) | 1190 (63·5%) |
| Overweight (n=3860) | 375 (21·9%) | 370 (17·9%) | 392 (19·4%) | 357 (20·7%) | 259 (19·6%) | 409 (21·6%) | 453 (21·9%) | 386 (20·5%) | 465 (23·1%) | 394 (21·0%) |
| Obese (n=1765) | 161 (9·4%) | 183 (8·9%) | 195 (9·7%) | 141 (8·2%) | 120 (9·1%) | 183 (9·7%) | 175 (8·5%) | 177 (9·4%) | 210 (10·4%) | 220 (11·7%) |

| | **Male partners (n=18,110)** | | | | | | | | | |
|---|---|---|---|---|---|---|---|---|---|---|
| **Years (n)** | **2010** (n=1536) | **2011** (n=1977) | **2012** (n=2143) | **2013** (n=1651) | **2014** (n=1264) | **2015** (n=1879) | **2016** (n=1968) | **2017** (n=1886) | **2018** (n=2028) | **2019** (n=1778) |
| **BMI (unadjusted)** | | | | | | | | | | |
| Mean (SD) | 25·7 (3·4) | 25·7 (3·5) | 25·8 (3·6) | 25·7 (3·6) | 25·7 (3·4) | 25·8 (3·8) | 25·8 (3·8) | 26·1 (3·9) | 26·0 (4·1) | 26·1 (4·0) |
| Median | 25·3 | 25·2 | 25·3 | 25·3 | 25·2 | 25·2 | 25·2 | 25·5 | 25·4 | 25·5 |

| | | | | | | | | | | |
|---|---|---|---|---|---|---|---|---|---|---|
| (Min, Max) | (17·2, 52·6) | (17·3, 46·4) | (15·8, 44·2) | (14·9, 58·8) | (16·4, 42·9) | (12·6, 56·8) | (12·3, 54·4) | (16·3, 59·9) | (12·4, 54·9) | (13·0, 57·1) |
| **Age** | | | | | | | | | | |
| Mean (SD) | 32·2 (5·9) | 32·1 (5·9) | 32·3 (5·7) | 32·2 (5·9) | 31·9 (5·6) | 32·1 (5·7) | 32·3 (5·7) | 32.3 (5·6) | 32·2 (5·6) | 32·7 (5·5) |
| Min, Max | 17·0, 58·0 | 15·0, 65·0 | 18·0, 64·0 | 18·0, 60·0 | 18·0, 61·0 | 19·0, 55·0 | 17·0, 63·0 | 17·0, 61·0 | 17·0, 57·0 | 20·0, 61·0 |
| **BMI categories (age-adjusted)** | | | | | | | | | | |
| Underweight (n=80) | 2 (0·1%) | 3 (0·2%) | 7 (0·3%) | 7 (0·4%) | 7 (0·6%) | 12 (0·6%) | 9 (0·5%) | 8 (0·4%) | 9 (0·4%) | 16 (0·9%) |
| Normal Weight (n=8203) | 713 (46·4%) | 933 (47·2%) | 973 (45·4%) | 764 (46·3%) | 585 (46·3%) | 856 (45·6%) | 923 (46·9%) | 788 (41·8%) | 894 (44·1%) | 774 (43·5%) |
| Overweight (n=7769) | 684 (44·5%) | 847 (42·8%) | 921 (43·0%) | 721 (43·7%) | 533 (42·2%) | 789 (42·0%) | 801 (40·7%) | 861 (45·7%) | 852 (42·0%) | 760 (42·7%) |
| Obese (n=2058) | 137 (8·9%) | 194 (9·8%) | 242 (11·3%) | 159 (9·6%) | 139 (11·0%) | 222 (11·8%) | 235 (11·9%) | 229 (12·1%) | 273 (13·5%) | 228 (12·8%) |

| Live births in Västerbotten by year (n=29,047) (16) | | | | | | | | | | |
|---|---|---|---|---|---|---|---|---|---|---|
| **Years** | **2010** | **2011** | **2012** | **2013** | **2014** | **2015** | **2016** | **2017** | **2018** | **2019** |
| **Number of live births** | 2891 | 2947 | 2835 | 2918 | 2770 | 2889 | 2995 | 2954 | 2914 | 2934 |

[a] column percentage.

**Table 2: Age-adjusted prevalence of underweight, normal weight, overweight and obesity by educational attainment, for women and men (n= 36,105) [a].**

| | Pregnant women (n=18,215) | | | | | | | | | |
|---|---|---|---|---|---|---|---|---|---|---|
| | Low education (n=6662) | | | | | | | | | |
| **Years (n)** | **2010** (n=666) | **2011** (n=783) | **2012** (n=740) | **2013** (n=630) | **2014** (n=452) | **2015** (n=689) | **2016** (n=738) | **2017** (n=644) | **2018** (n=717) | **2019** (n=603) |
| **BMI categories** | | | | | | | | | | |
| Underweight (n=329) | 40 (6·0%) [b] | 41 (5·2%) | 40 (5·4%) | 39 (6·2%) | 16 (3·5%) | 39 (5·7%) | 33 (4·5%) | 28 (4·3%) | 28 (3·9%) | 25 (4·1%) |
| Normal Weight (n=3968) | 377 (56·6%) | 487 (62·2%) | 438 (59·2%) | 389 (61·7%) | 289 (63·9%) | 404 (58·6%) | 437 (59·2%) | 387 (60·1%) | 413 (57·6%) | 347 (57·5%) |
| Overweight (n=1487) | 166 (24·9%) | 148 (18·9%) | 155 (20·9%) | 135 (21·4%) | 96 (21·2%) | 155 (22·5%) | 184 (24·9%) | 143 (22·2%) | 177 (24·7%) | 128 (21·2%) |
| Obese (n=878) | 83 (12·5%) | 107 (13·7%) | 107 (14·5%) | 67 (10·6%) | 51 (11·3%) | 91 (13·2%) | 84 (11·4%) | 86 (13·4%) | 99 (13·8%) | 103 (17·1%) |
| | High education (n=11,553) | | | | | | | | | |

| Years (n) | 2010 (n=1016) | 2011 (n=1236) | 2012 (n=1249) | 2013 (n=1053) | 2014 (n=834) | 2015 (n=1178) | 2016 (n=1296) | 2017 (n=1199) | 2018 (n=1259) | 2019 (n=1233) |
|---|---|---|---|---|---|---|---|---|---|---|
| **BMI categories** | | | | | | | | | | |
| Underweight (n=377) | 37 (3·6%) | 48 (3·9%) | 40 (3·2%) | 41 (3·9%) | 27 (3·2%) | 31 (2·6%) | 36 (2·8%) | 46 (3·8%) | 33 (2·6%) | 38 (3·1%) |
| Normal Weight (n=8039) | 705 (69·4%) | 909 (73·5%) | 893 (71·5%) | 731 (69·4%) | 585 (70·1%) | 813 (69·0%) | 911 (70·3%) | 828 (69·1%) | 845 (67·1%) | 819 (66·4%) |
| Overweight (n=2294) | 201 (19·8%) | 209 (16·9%) | 231 (18·5%) | 210 (19·9%) | 156 (18·7%) | 247 (21·0%) | 262 (20·2%) | 240 (20·0%) | 277 (22·0%) | 261 (21·2%) |
| Obese (n=843) | 73 (7·2%) | 70 (5·7%) | 85 (6·8%) | 71 (6·7%) | 66 (7·9%) | 87 (7·4%) | 87 (6·7%) | 85 (7·1%) | 104 (8·3%) | 115 (9·3%) |

| | Male partners (n=17,890) | | | | | | | | | |
|---|---|---|---|---|---|---|---|---|---|---|
| | Low education (n=9341) | | | | | | | | | |
| **Years (n)** | **2010** (n=820) | **2011** (n=1029) | **2012** (n=1113) | **2013** (n=852) | **2014** (n=640) | **2015** (n=985) | **2016** (n=1000) | **2017** (n=978) | **2018** (n=1063) | **2019** (n=861) |

| | | | | | | | | | | |
|---|---|---|---|---|---|---|---|---|---|---|
| **BMI categories** | | | | | | | | | | |
| Underweight (n=41) | 1 (0·1%) | 1 (0·1%) | 4 (0·4%) | 3 (0·4%) | 2 (0·3%) | 5 (0·5%) | 8 (0·8%) | 6 (0·6%) | 4 (0·4%) | 7 (0·8%) |
| Normal Weight (n=3748) | 339 (41·3%) | 447 (43·4%) | 441 (39·6%) | 360 (42·3%) | 264 (41·3%) | 399 (40·5%) | 410 (41·0%) | 363 (37·1%) | 401 (37·7%) | 324 (37·6%) |
| Overweight (n=4223) | 388 (47·3%) | 448 (43·5%) | 503 (45·2%) | 395 (46·4%) | 291 (45·5%) | 436 (44·3%) | 436 (43·6%) | 456 (46·6%) | 479 (45·1%) | 391 (45·4%) |
| Obese (n=1329) | 92 (11·2%) | 133 (12·9%) | 165 (14·8%) | 94 (11·0%) | 83 (13·0%) | 145 (14·7%) | 146 (14·6%) | 153 (15·6%) | 179 (16·8%) | 139 (16·1%) |

| | | | | | | | | | | |
|---|---|---|---|---|---|---|---|---|---|---|
| | | | | | **High education (n=8549)** | | | | | |
| **Years (n)** | **2010** (N=684) | **2011** (N=927) | **2012** (N=998) | **2013** (N=767) | **2014** (N=607) | **2015** (N=884) | **2016** (N=952) | **2017** (N=886) | **2018** (N=939) | **2019** (N=905) |
| **BMI categories** | | | | | | | | | | |
| Underweight (n=39) | 1 (0·1%) | 2 (0·2%) | 3 (0·3%) | 4 (0·5%) | 5 (0·8%) | 7 (0·8%) | 1 (0·1%) | 2 (0·2%) | 5 (0·5%) | 9 (1·0%) |
| Normal Weight | 358 (52·3%) | 477 (51·5%) | 517 (51·8%) | 395 (51·5%) | 314 (51·7%) | 450 (50·9%) | 509 (53·5%) | 418 (47·2%) | 481 (51·2%) | 442 (48·8%) |

| | | | | | | | | | | |
|---|---|---|---|---|---|---|---|---|---|---|
| (n=4361) | | | | | | | | | | |
| Overweight (n=3456) | 283 (41·4%) | 391 (42·2%) | 405 (40·6%) | 309 (40·3%) | 236 (38·9%) | 351 (39·7%) | 356 (37·4%) | 396 (44·7%) | 363 (38·7%) | 366 (40·4%) |
| Obese (n=693) | 42 (6·1%) | 57 (6·1%) | 73 (7·3%) | 59 (7·7%) | 52 (8·6%) | 76 (8·6%) | 86 (9·0%) | 70 (7·9%) | 90 (9·6%) | 88 (9·7%) |

[a] 353 with missing education for pregnant women and 220 with missing education for male partners / [b] column percentage.

**Table 3: Prevalence ratios and differences in overweight and obesity (for women and men), and underweight (for women), comparing groups with low education to groups with high education (n= 36,105).**

| | | Pregnant women (n=18,215) | | | | | | | | | |
|---|---|---|---|---|---|---|---|---|---|---|---|
| | **Year** | **2010** | **2011** | **2012** | **2013** | **2014** | **2015** | **2016** | **2017** | **2018** | **2019** |
| **Underweight** | **PR** [CI] | **1·65** [1·07-2·56] | **1·35** [0·89-2·02] | **1·69** [1·10-2·60] | **1·59** [1·03-2·44] | **1·09** [0·58-1·98] | **2·15** [1·36-3·44] | **1·61** [1·01-2·56] | **1·13** [0·71-1·78] | **1·49** [0·90-2·44] | **1·35** [0·81-2·19] |
| | p-value | 0·025 | 0·15 | 0·017 | 0·033 | 0·773 | 0·001 | 0·044 | 0·594 | 0·115 | 0·241 |
| | **PD** [CI] | **2·4** [0·3-4·6] | **1·4** [-0·5-3·3] | **2·2** [0·4-4·2] | **2·3** [0·2-4·6] | **0·3** [-1·7-2·5] | **3·0** [1·2-5·1] | **1·7** [0·0-3·5] | **0·5** [-1·3-2·5] | **1·3** [-0·3-3·1] | **1·1** [-0·7-3·1] |
| | p-value | 0·03 | 0·162 | 0·023 | 0·042 | 0·776 | 0·002 | 0·056 | 0·601 | 0·132 | 0·262 |
| **Overweight** | **PR** [CI] | **1·26** [1·05-1·51] | **1·12** [0·92-1·35] | **1·13** [0·94-1·36] | **1·07** [0·88-1·30] | **1·14** [0·90-1·42] | **1·07** [0·90-1·28] | **1·23** [1·04-1·45] | **1·11** [0·92-1·33] | **1·12** [0·95-1·32] | **1·00** [0·83-1·21] |
| | p-value | 0·012 | 0·252 | 0·18 | 0·464 | 0·273 | 0·437 | 0·013 | 0·268 | 0·171 | 0·977 |

| | | | | | | | | | | | |
|---|---|---|---|---|---|---|---|---|---|---|---|
| | **PD** | **5·1** | **2·0** | **2·5** | **1·5** | **2·5** | **1·5** | **4·7** | **2·2** | **2·7** | **0·1** |
| | [CI] | [1·1-9·3] | [-1·4-5·5] | [-1·1-6·1] | [-2·5-5·5] | [-2·0-7·2] | [-2·3-5·5] | [0·9-8·6] | [-1·7-6·2] | [-1·2-6·6] | [-3·9-4·1] |
| | p-value | 0·014 | 0·257 | 0·187 | 0·468 | 0·281 | 0·441 | 0·015 | 0·275 | 0·177 | 0·977 |
| **Obesity** | **PR** | **1·73** | **2·41** | **2·12** | **1·58** | **1·43** | **1·79** | **1·70** | **1·88** | **1·67** | **1·83** |
| | [CI] | [1·29-2·34] | [1·81-3·23] | [1·62-2·79] | [1·14-2·17] | [1·00-2·01] | [1·35-2·36] | [1·27-2·26] | [1·42-2·50] | [1·29-2·17] | [1·43-2·35] |
| | p-value | <0·001 | <0·001 | <0·001 | 0·005 | 0·045 | <0·001 | <0·001 | <0·001 | <0·001 | <0·001 |
| | **PD** | **5·3** | **8·0** | **7·7** | **3·9** | **3·4** | **5·8** | **4·7** | **6·3** | **5·5** | **7·8** |
| | [CI] | [2·4-8·3] | [5·3-10·8] | [4·8-10·6] | [1·1-6·8] | [-0·0-6·9] | [3·0-8·8] | [2·1-7·4] | [3·3-9·4] | [2·7-8·6] | [4·4-11·3] |
| | p-value | <0·001 | <0·001 | <0·001 | 0·007 | 0·055 | <0·001 | 0·001 | <0·001 | <0·001 | <0·001 |

| | **Male partners (n=17,890)** | | | | | | | | | | |
|---|---|---|---|---|---|---|---|---|---|---|---|
| | **Year** | **2010** | **2011** | **2012** | **2013** | **2014** | **2015** | **2016** | **2017** | **2018** | **2019** |

| | | | | | | | | | | | |
|---|---|---|---|---|---|---|---|---|---|---|---|
| **Overweight** | **PR** [CI] | **1·14** [1·02-1·28] | **1·03** [0·93-1·14] | **1·11** [1·01-1·23] | **1·15** [1·03-1·29] | **1·17** [1·03-1·33] | **1·11** [1·00-1·24] | **1·17** [1·05-1·30] | **1·04** [0·94-1·15] | **1·17** [1·05-1·29] | **1·12** [1·01-1·25] |
| | p-value | 0·022 | 0·545 | 0·033 | 0·014 | 0·019 | 0·047 | 0·005 | 0·404 | 0·004 | 0·035 |
| | **PD** [CI] | **5·9** [0·9-11·0] | **1·4** [-3·0-5·7] | **4·6** [0·4-8·8] | **6·1** [1·2-10·9] | **6·6** [1·1-12·0] | **4·6** [0·1-9·0] | **6·2** [1·9-10·5] | **1·9** [-2·6-6·5] | **6·4** [2·1-10·7] | **5·0** [0·4-9·6] |
| | p-value | 0·021 | 0·544 | 0·032 | 0·014 | 0·018 | 0·046 | 0·005 | 0·403 | 0·004 | 0·035 |
| **Obesity** | **PR** [CI] | **1·83** [1·30-2·62] | **2·10** [1·57-2·85] | **2·03** [1·57-2·65] | **1·43** [1·06-1·97] | **1·51** [1·09-2·12] | **1·71** [1·32-2·24] | **1·62** [1·26-2·09] | **1·98** [1·52-2·60] | **1·76** [1·39-2·24] | **1·66** [1·30-2·14] |
| | p-value | 0·001 | <0·001 | <0·001 | 0·023 | 0·013 | <0·001 | <0·001 | <0·001 | <0·001 | <0·001 |
| | **PD** [CI] | **5·1** [2·3-7·9] | **6·8** [4·2-9·4] | **7·5** [4·9-10·2] | **3·3** [0·5-6·2] | **4·4** [1·0-7·9] | **6·1** [3·2-9·0] | **5·6** [2·7-8·4] | **7·7** [4·9-10·6] | **7·3** [4·3-10·2] | **6·4** [3·3-9·6] |
| | p-value | <0·001 | <0·001 | <0·001 | 0·02 | 0·012 | <0·001 | <0·001 | <0·001 | <0·001 | <0·001 |

PR = Prevalence Ratio; PD = Prevalence Difference (in percentage points); CI = 95% confidence interval; the reference group is « High education ».

Prevalence difference = (Prevalence in low educated - Prevalence in high educated).

**Table 4: Unadjusted prevalence of underweight, normal weight, overweight and obesity by year, among pregnant women and male partners (n=36,678).**

| | Pregnant women (n=18,568) | | | | | | | | | |
|---|---|---|---|---|---|---|---|---|---|---|
| **Years (n)** | **2010** (n=1716) | **2011** (n=2063) | **2012** (n=2020) | **2013** (n=1725) | **2014** (n=1321) | **2015** (n=1891) | **2016** (n=2067) | **2017** (n=1880) | **2018** (n=2011) | **2019** (n=1874) |
| **BMI categories** | | | | | | | | | | |
| Underweight (n=740) | 77 (4.5%) [a] | 93 (4.5%) | 80 (4.0%) | 82 (4.8%) | 49 (3.7%) | 74 (3.9%) | 74 (3.6%) | 75 (4.0%) | 65 (3.2%) | 71 (3.8%) |
| Normal Weight (n= 12,177) | 1101 (64.2%) | 1411 (68.4%) | 1356 (67.1%) | 1138 (66.0%) | 890 (67.4%) | 1227 (64.9%) | 1368 (66.2%) | 1240 (66.0%) | 1262 (62.8%) | 1184 (63.2%) |
| Overweight (n=3841) | 370 (21.6%) | 373 (18.1%) | 382 (18.9%) | 357 (20.7%) | 261 (19.8%) | 402 (21.3%) | 446 (21.6%) | 381 (20.3%) | 473 (23.5%) | 396 (21.1%) |
| Obese (n=1810) | 168 (9.8%) | 186 (9.0%) | 202 (10.0%) | 148 (8.6%) | 121 (9.2%) | 188 (9.9%) | 179 (8.7%) | 184 (9.8%) | 211 (10.5%) | 223 (11.9%) |
| | **Male partners (n=18,110)** | | | | | | | | | |

| Years (n) | 2010 (n=1536) | 2011 (n=1977) | 2012 (n=2143) | 2013 (n=1651) | 2014 (n=1264) | 2015 (n=1879) | 2016 (n=1968) | 2017 (n=1886) | 2018 (n=2028) | 2019 (n=1778) |
|---|---|---|---|---|---|---|---|---|---|---|
| **BMI categories** | | | | | | | | | | |
| Underweight (n=89) | 3 (0.2%) | 5 (0.3%) | 7 (0.3%) | 6 (0.4%) | 9 (0.7%) | 13 (0.7%) | 12 (0.6%) | 7 (0.4%) | 11 (0.5%) | 16 (0.9%) |
| Normal Weight (n=8254) | 706 (46.0%) | 926 (46.8%) | 991 (46.2%) | 766 (46.4%) | 593 (46.9%) | 873 (46.5%) | 928 (47.2%) | 806 (42.7%) | 905 (44.6%) | 760 (42.7%) |
| Overweight (n=7706) | 683 (44.5%) | 853 (43.1%) | 898 (41.9%) | 715 (43.3%) | 524 (41.5%) | 768 (40.9%) | 799 (40.6%) | 844 (44.8%) | 846 (41.7%) | 776 (43.6%) |
| Obese (n=2061) | 144 (9.4%) | 193 (9.8%) | 247 (11.5%) | 164 (9.9%) | 138 (10.9%) | 225 (12.0%) | 229 (11.6%) | 229 (12.1%) | 266 (13.1%) | 226 (12.7%) |

[a] column percentage.

**Table 5: Results from multinomial logistic regression of age-adjusted BMI categories on year for pregnant women (n=18,568).**

| | **Underweight** [a] | | | **Overweight** [a] | | | **Obesity** [a] | | |
|---|---|---|---|---|---|---|---|---|---|
| **Year** | OR | [95% CI] | p-value | OR | [95% CI] | p-value | OR | [95% CI] | p-value |
| **2010** | Ref | | | Ref | | | Ref | | |
| **2011** | 0.93 | [0.68-1.27] | 0.646 | 0.77 | [0.65-0.90] | 0.002 | 0.88 | [0.71-1.11] | 0.285 |
| **2012** | 0.86 | [0.62-1.18] | 0.352 | 0.85 | [0.72-1.00] | 0.055 | 0.99 | [0.79-1.24] | 0.916 |
| **2013** | 1.03 | [0.74-1.42] | 0.876 | 0.92 | [0.78-1.08] | 0.309 | 0.84 | [0.66-1.07] | 0.166 |
| **2014** | 0.74 | [0.51-1.07] | 0.109 | 0.85 | [0.71-1.02] | 0.079 | 0.92 | [0.71-1.18] | 0.504 |
| **2015** | 0.82 | [0.58-1.14] | 0.232 | 0.98 | [0.83-1.15] | 0.796 | 1.02 | [0.81-1.28] | 0.864 |
| **2016** | 0.74 | [0.53-1.04] | 0.080 | 0.97 | [0.83-1.14] | 0.744 | 0.88 | [0.70-1.10] | 0.257 |
| **2017** | 0.89 | [0.64-1.23] | 0.482 | 0.92 | [0.78-1.08] | 0.291 | 0.98 | [0.78-1.23] | 0.848 |
| **2018** | 0.69 | [0.49-0.97] | 0.032 | 1.07 | [0.92-1.26] | 0.384 | 1.13 | [0.91-1.41] | 0.283 |
| **2019** | 0.84 | [0.60-1.18] | 0.316 | 0.97 | [0.83-1.15] | 0.751 | 1.27 | [1.02-1.58] | 0.035 |

[a] normal weight is the reference group for the outcome.

**Table 6: Results from multinomial logistic regression of age-adjusted BMI categories on year for male partners (n=18,110).**

| | **Underweight [a]** | | | **Overweight [a]** | | | **Obesity [a]** | | |
|---|---|---|---|---|---|---|---|---|---|
| **Year** | OR | [95% CI] | p-value | OR | [95% CI] | p-value | OR | [95% CI] | p-value |
| **2010** | Ref | | | Ref | | | Ref | | |
| **2011** | 1.14 | [0.19-6.85] | 0.884 | 0.95 | [0.82-1.09] | 0.441 | 1.08 | [0.85-1.38] | 0.518 |
| **2012** | 2.56 | [0.53-12.34] | 0.242 | 0.99 | [0.86-1.13] | 0.850 | 1.29 | [1.03-1.63] | 0.028 |
| **2013** | 3.26 | [0.68-15.73] | 0.141 | 0.98 | [0.85-1.14] | 0.826 | 1.08 | [0.84-1.39] | 0.532 |
| **2014** | 4.26 | [0.88-20.56] | 0.071 | 0.95 | [0.81-1.11] | 0.521 | 1.24 | [0.95-1.60] | 0.109 |
| **2015** | 4.99 | [1.11-22.34] | 0.036 | 0.96 | [0.83-1.11] | 0.583 | 1.35 | [1.07-1.71] | 0.012 |
| **2016** | 3.47 | [0.75-16.08] | 0.112 | 0.90 | [0.79-1.04] | 0.164 | 1.33 | [1.05-1.67] | 0.018 |
| **2017** | 3.61 | [0.77-17.05] | 0.105 | 1.14 | [0.99-1.31] | 0.074 | 1.51 | [1.20-1.91] | 0.001 |
| **2018** | 3.58 | [0.77-16.61] | 0.103 | 0.99 | [0.86-1.14] | 0.927 | 1.59 | [1.27-2.00] | <0.001 |
| **2019** | 7.35 | [1.69-32.06] | 0.008 | 1.02 | [0.89-1.18] | 0.753 | 1.53 | [1.21-1.94] | <0.001 |

[a] normal weight is the reference group for the outcome.

**Table 7: Results from multinomial regression of age-adjusted BMI categories on year, education, and year*education for pregnant women (n=18,215).**

| | **Underweight** [a] | | | **Overweight** [a] | | | **Obesity** [a] | | |
|---|---|---|---|---|---|---|---|---|---|
| | OR | [95% CI] | p-value | OR | [95% CI] | p-value | OR | [95% CI] | p-value |
| **Year** | | | | | | | | | |
| **2010** | Ref | | | Ref | | | Ref | | |
| **2011** | 1.01 | [0.65-1.56] | 0.978 | 0.81 | [0.65-1.00] | 0.052 | 0.74 | [0.53-1.05] | 0.090 |
| **2012** | 0.85 | [0.54-1.35] | 0.498 | 0.91 | [0.73-1.12] | 0.371 | 0.92 | [0.66-1.28] | 0.615 |
| **2013** | 1.07 | [0.68-1.69] | 0.775 | 1.01 | [0.81-1.25] | 0.946 | 0.94 | [0.67-1.32] | 0.714 |
| **2014** | 0.88 | [0.53-1.46] | 0.620 | 0.94 | [0.74-1.18] | 0.579 | 1.09 | [0.77-1.55] | 0.631 |
| **2015** | 0.73 | [0.45-1.18] | 0.199 | 1.07 | [0.86-1.32] | 0.556 | 1.03 | [0.75-1.43] | 0.844 |
| **2016** | 0.75 | [0.47-1.20] | 0.236 | 1.01 | [0.82-1.24] | 0.935 | 0.92 | [0.67-1.28] | 0.627 |
| **2017** | 1.06 | [0.68-1.65] | 0.802 | 1.02 | [0.82-1.26] | 0.879 | 0.99 | [0.71-1.38] | 0.959 |
| **2018** | 0.74 | [0.46-1.20] | 0.227 | 1.15 | [0.93-1.41] | 0.187 | 1.19 | [0.87-1.63] | 0.283 |
| **2019** | 0.88 | [0.56-1.41] | 0.603 | 1.12 | [0.91-1.38] | 0.298 | 1.36 | [0.99-1.85] | 0.054 |

| | | | | | | | | | |
|---|---|---|---|---|---|---|---|---|---|
| **Education** | | | | | | | | | |
| **High education** | Ref | | | Ref | | | Ref | | |
| **Low education** | 2.02 | [1.27-3.22] | 0.003 | 1.54 | [1.21-1.96] | <0.001 | 2.13 | [1.52-2.98] | <0.001 |
| **Year*Education** | | | | | | | | | |
| **2011*Low education** | 0.79 | [0.42-1.49] | 0.463 | 0.86 | [0.61-1.20] | 0.367 | 1.34 | [0.84-2.14] | 0.216 |
| **2012*Low education** | 1.01 | [0.53-1.93] | 0.980 | 0.89 | [0.63-1.24] | 0.478 | 1.21 | [0.76-1.91] | 0.419 |
| **2013*Low education** | 0.88 | [0.46-1.69] | 0.711 | 0.78 | [0.55-1.11] | 0.164 | 0.83 | [0.51-1.36] | 0.469 |
| **2014*Low education** | 0.59 | [0.27-1.30] | 0.193 | 0.81 | [0.55-1.18] | 0.264 | 0.74 | [0.44-1.23] | 0.245 |
| **2015*Low education** | 1.25 | [0.64-2.45] | 0.512 | 0.82 | [0.58-1.14] | 0.239 | 0.99 | [0.62-1.57] | 0.966 |
| **2016*Low education** | 0.95 | [0.48-1.85] | 0.870 | 0.95 | [0.68-1.31] | 0.748 | 0.95 | [0.59-1.51] | 0.818 |
| **2017*Low education** | 0.64 | [0.33-1.26] | 0.199 | 0.83 | [0.59-1.16] | 0.268 | 1.02 | [0.64-1.63] | 0.940 |
| **2018*Low education** | 0.86 | [0.43-1.72] | 0.667 | 0.85 | [0.61-1.17] | 0.319 | 0.92 | [0.58-1.44] | 0.704 |
| **2019*Low education** | 0.77 | [0.38-1.54] | 0.458 | 0.75 | [0.53-1.06] | 0.100 | 0.99 | [0.64-1.56] | 0.980 |

[a] normal weight is the reference group for the outcome.

**Table 8: Results from multinomial regression of age-adjusted BMI categories on year, education, and year*education for male partners (n=17,890).**

| | **Underweight** [a] | | | **Overweight** [a] | | | **Obesity** [a] | | |
|---|---|---|---|---|---|---|---|---|---|
| | OR | [95% CI] | p-value | OR | [95% CI] | p-value | OR | [95% CI] | p-value |
| **Year** | | | | | | | | | |
| **2010** | Ref | | | Ref | | | Ref | | |
| **2011** | 1.50 | [0.14-16.62] | 0.741 | 1.04 | [0.84-1.27] | 0.729 | 1.02 | [0.67-1.55] | 0.932 |
| **2012** | 2.08 | [0.22-20.05] | 0.527 | 0.99 | [0.81-1.21] | 0.930 | 1.20 | [0.80-1.80] | 0.367 |
| **2013** | 3.63 | [0.40-32.59] | 0.250 | 0.99 | [0.80-1.23] | 0.924 | 1.27 | [0.84-1.94] | 0.261 |
| **2014** | 5.70 | [0.66-49.06] | 0.113 | 0.95 | [0.76-1.20] | 0.667 | 1.41 | [0.91-2.18] | 0.119 |
| **2015** | 5.57 | [0.68-45.47] | 0.109 | 0.99 | [0.80-1.22] | 0.900 | 1.44 | [0.96-2.15] | 0.075 |
| **2016** | 0.70 | [0.04-11.28] | 0.804 | 0.88 | [0.72-1.09] | 0.245 | 1.44 | [0.97-2.13] | 0.069 |
| **2017** | 1.71 | [0.15-18.97] | 0.661 | 1.20 | [0.97-1.48] | 0.088 | 1.43 | [0.95-2.15] | 0.087 |
| **2018** | 3.72 | [0.43-31.99] | 0.231 | 0.95 | [0.78-1.17] | 0.661 | 1.59 | [1.08-2.36] | 0.019 |
| **2019** | 7.29 | [0.92-57.81] | 0.060 | 1.05 | [0.85-1.29] | 0.663 | 1.70 | [1.15-2.51] | 0.008 |

| | | | | | | | | | |
|---|---|---|---|---|---|---|---|---|---|
| **Education** | | | | | | | | | |
| **High education** | Ref | | | Ref | | | Ref | | |
| **Low education** | 1.06 | [0.07-16.95] | 0.969 | 1.45 | [1.17-1.79] | 0.001 | 2.31 | [1.56-3.43] | <0.001 |
| **Year*Education** | | | | | | | | | |
| **2011*Low education** | 0.51 | [0.01-19.87] | 0.716 | 0.84 | [0.64-1.12] | 0.243 | 1.08 | [0.64-1.81] | 0.781 |
| **2012*Low education** | 1.48 | [0.06-34.76] | 0.808 | 1.01 | [0.76-1.33] | 0.969 | 1.15 | [0.70-1.88] | 0.592 |
| **2013*Low education** | 0.78 | [0.03-18.31] | 0.877 | 0.97 | [0.72-1.30] | 0.834 | 0.76 | [0.44-1.28] | 0.301 |
| **2014*Low education** | 0.45 | [0.02-11.37] | 0.628 | 1.01 | [0.74-1.39] | 0.937 | 0.82 | [0.47-1.42] | 0.481 |
| **2015*Low education** | 0.76 | [0.04-15.42] | 0.860 | 0.97 | [0.72-1.29] | 0.823 | 0.93 | [0.56-1.53] | 0.777 |
| **2016*Low education** | 9.40 | [0.29-302.33] | 0.206 | 1.05 | [0.79-1.40] | 0.738 | 0.91 | [0.56-1.49] | 0.711 |
| **2017*Low education** | 3.27 | [0.13-80.83] | 0.469 | 0.92 | [0.69-1.22] | 0.551 | 1.09 | [0.66-1.80] | 0.743 |
| **2018*Low education** | 0.91 | [0.04-19.66] | 0.951 | 1.09 | [0.82-1.45] | 0.541 | 1.03 | [0.63-1.68] | 0.901 |
| **2019*Low education** | 1.00 | [0.05-19.19] | 0.998 | 1.01 | [0.75-1.35] | 0.965 | 0.93 | [0.57-1.53] | 0.780 |

[a] normal weight is the reference group for the outcome.
